# Responses of pyramidal cell somata and apical dendrites in mouse visual cortex over multiple days

**DOI:** 10.1038/s41597-023-02214-y

**Published:** 2023-05-17

**Authors:** Colleen J. Gillon, Jérôme A. Lecoq, Jason E. Pina, Ruweida Ahmed, Yazan N. Billeh, Shiella Caldejon, Peter Groblewski, Timothy M. Henley, India Kato, Eric Lee, Jennifer Luviano, Kyla Mace, Chelsea Nayan, Thuyanh V. Nguyen, Kat North, Jed Perkins, Sam Seid, Matthew T. Valley, Ali Williford, Yoshua Bengio, Timothy P. Lillicrap, Joel Zylberberg, Blake A. Richards

**Affiliations:** 1 Department of Cell & Systems Biology, University of Toronto, Toronto, Ontario, Canada; 2 Department of Biological Sciences, University of Toronto Scarborough, Toronto, Ontario, Canada; 3 Mila, Montréal, Québec, Canada; 4 Allen Institute, MindScope Program, Seattle, WA USA; 5 Department of Physics and Astronomy, York University, Toronto, Ontario Canada; 6 Centre for Vision Research, York University, Toronto, Ontario Canada; 7 Département d’informatique et de recherche opérationnelle, Université de Montréal, Montréal, Québec Canada; 8 Learning in Machines and Brains Program, Canadian Institute for Advanced Research, Toronto, Ontario Canada; 9 DeepMind, Inc, London, UK; 10 Centre for Computation, Mathematics and Physics in the Life Sciences and Experimental Biology, University College London, London, UK; 11 Vector Institute for Artificial Intelligence, Toronto, Ontario Canada; 12 School of Computer Science, McGill University, Montréal, Québec Canada; 13 Department of Neurology & Neurosurgery, McGill University, Montréal, Québec Canada; 14 Montreal Neurological Institute, McGill University, Montréal, Québec Canada

**Keywords:** Sensory processing, Striate cortex

## Abstract

The apical dendrites of pyramidal neurons in sensory cortex receive primarily top-down signals from associative and motor regions, while cell bodies and nearby dendrites are heavily targeted by locally recurrent or bottom-up inputs from the sensory periphery. Based on these differences, a number of theories in computational neuroscience postulate a unique role for apical dendrites in learning. However, due to technical challenges in data collection, little data is available for comparing the responses of apical dendrites to cell bodies over multiple days. Here we present a dataset collected through the Allen Institute Mindscope’s OpenScope program that addresses this need. This dataset comprises high-quality two-photon calcium imaging from the apical dendrites and the cell bodies of visual cortical pyramidal neurons, acquired over multiple days in awake, behaving mice that were presented with visual stimuli. Many of the cell bodies and dendrite segments were tracked over days, enabling analyses of how their responses change over time. This dataset allows neuroscientists to explore the differences between apical and somatic processing and plasticity.

## Background & Summary

Pyramidal neurons are the primary excitatory neurons in the neocortex, and are thus of major importance in sensation, behaviour, and cognition. Pyramidal neurons have a striking anatomical structure: while their cell bodies lie at different depths within the cortex, they each have a long apical dendrite that extends, in many cases, up to the cortical surface. The inputs to these apical dendrites are typically from neurons in other downstream cortical regions or associative thalamic regions^1–3^, in contrast to the basal dendrites which lie near the soma and are heavily innervated by inputs from nearby neurons within the same cortical region, or from sensory subcortical structures like the primary thalamic nuclei^1,2^. Moreover, there are profound physiological differences between the apical and basal dendrites related to the distribution of ion channel and synaptic receptor types. For example, the apical dendrites have more voltage-gated calcium channels that make them more prone to developing plateau potentials in response to strong synaptic inputs^4–6^. These anatomical and physiological differences suggest that inputs to the apical versus basal dendrites might serve different computational roles, which has motivated the development of many computational models of learning and inference in neocortical circuits^7–9^.

Despite the strong interest in how apical dendrites contribute to learning and inference, there have, to-date, been few experimental datasets that can speak to these myriad theoretical models. This limitation primarily arises from the significant challenge of obtaining high-resolution chronic recordings from the apical dendrites of multiple cells in awake behaving animals. Their small diameter, e.g. on the order of 1*μm*, means that there is a relatively low signal to noise ratio (SNR) when imaging these cellular processes, and resolving them necessitates a high spatial resolution. Motion artifacts due to the animal’s locomotion, heartbeat, whisking, or other movements, add to this challenge. Segmenting microscopy data to identify individual dendritic segments, and removing background sources is also a challenge. Finally, all of these challenges conspire to make it difficult to identify the same dendritic segments in recordings from the same animal on different days. But, this matching is necessary for tracking any changes (due to learning, homeostasis^10,11^, or other processes) in the signals observed at these dendritic segments.

**Fig. 1:**
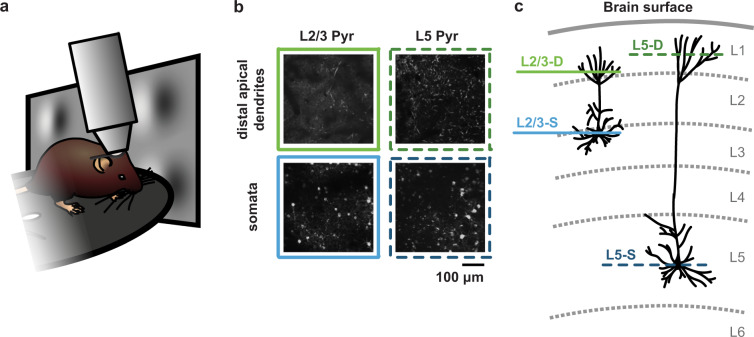
(**a**) Illustration of experimental setup. (**b**) Example images from the four imaging planes recorded. (**c**) Illustration of the location in the cortical laminae of each imaging plane.

To fill this gap in the range of datasets available, we leveraged the unique capabilities and thorough quality control pipeline of the Allen Brain Observatory at the Allen Institute. This enabled us to record from the apical dendrites (in cortical layer 1) and somata of pyramidal cells in mouse visual cortex, with the same imaging planes recorded over 3 different days (Fig. 1). During these recording sessions, animals were exposed to visual stimuli that were either consistent, or inconsistent, with those that they experienced during the week of habituation they underwent prior to the recording sessions. We presented these stimuli because many of the theories of learning in the neocortex postulate a special role for inconsistent stimuli^12^. By segmenting the data in each plane into regions of interest (ROIs), and registering these ROIs across recording days, we were able to identify single ROIs that were present in each day’s recording. This enabled us to track the location of individual apical dendrite segments or somata over the 3 days. Finally, we repeated these experiments in two different mouse lines: the Cux2-CreERT2;Camk2a-tTA;Ai93 line, where L2/3 pyramidal cells expressed the calcium indicator, and the Rbp4-Cre_KL100;Camk2a-tTA;Ai93 line, where L5 pyramidal cells expressed the calcium indicator. In addition to the neural data, we collected pupil position and diameter, as well as locomotion data during the recordings.

In this report, we provide an overview of the above-described experimental data^13^ and scripts to perform some basic analyses, both of which are freely available. The data format and scripts have all been designed to be as easy as possible for other groups to access and use. We hope, and anticipate, that other scientists can expand on these analyses, and that this resource will help the community to determine the role of pyramidal cell apical dendrites in sensory processing and learning.

## Methods

### Experimental animals and calcium imaging

**Fig. 2:**
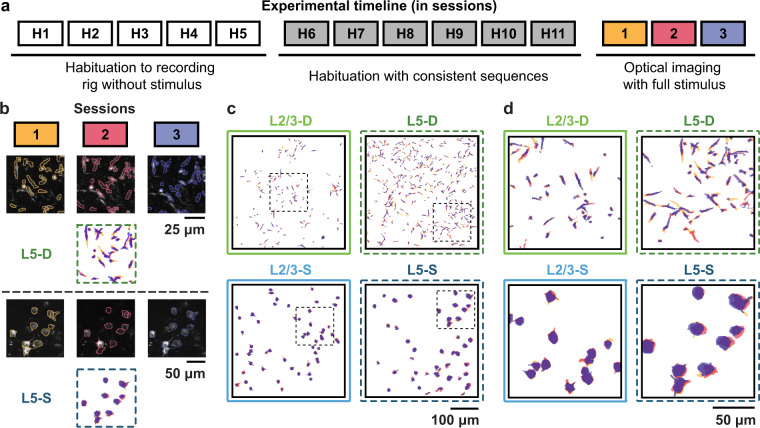
(**a**) Illustration of experimental timeline. (**b**) Example segmented calcium images across sessions, one from a dendritic plane, one from a somatic plane. (**c**) Full field examples of tracked ROIs in each of the imaging planes across sessions. Colours for each session same as those in a. & b. (**d**) Close-ups of the tracked ROIs from c. (areas indicated by black dotted squares in c.).

The dataset presented in this paper^13^ was collected as part of the Allen Institute Mindscope’s OpenScope initiative^14^. All animal procedures were approved by the Institutional Animal Care and Use Committee (IACUC) at the Allen Institute, under protocol 1801. Two transgenic mouse lines (Cux2-CreERT2;Camk2a-tTA;Ai93 and Rbp4-Cre_KL100;Camk2a-tTA;Ai93) were used to drive expression of GCaMP6f in layer 2/3 and layer 5 pyramidal neurons, respectively. Mice first underwent cranial window surgery, following which they were housed in cages individually and maintained on a reverse dark-light cycle with experiments conducted during the dark phase. Mice were then habituated over two weeks to head fixation on a running disc, with the visual stimulus presentation being added the second week (see below for detailed descriptions of the visual stimuli). Following habituation, they underwent three 70-minute optical imaging sessions within a span of three to six days, with no more than one session occurring per day (Fig. 2a). For each mouse, retinotopic mapping was performed under anaesthesia using intrinsic signal imaging (ISI) (for more details, see^15^). This enabled the two-photon calcium imaging recordings to be targeted precisely to the same area across mice, namely the retinotopic center of primary visual cortex (VisP). For each mouse, two-photon calcium imaging was performed in either the cell body layer for somatic recordings (175 *μ*m depth for layer 2/3 and 375 *μ*m depth for layer 5) or in cortical layer 1 for distal apical dendritic recordings (50–75 *μ*m depth for layer 2/3 and 20 *μ*m depth for layer 5) across all optical imaging sessions. In order to reduce Z-drift during imaging sessions, the cranial window pushes gently against the surface of the brain. This leads to slight compression of the brain, and is why our L5 somata, for example, were recorded at a shallower depth than might otherwise be expected in mouse VisP. 13 mice in total underwent imaging (L2/3-D: *n* = 3, L2/3-S: *n* = 3, L5-D: *n* = 4, L5-S: *n* = 3) with at least three optical imaging sessions recorded in each (see Tables 1, 2). Additional details on the Cre lines, surgery, habituation, and quality control can be found in previously published work from the Allen Institute^15^. In particular, supplementary figs. 12–19 of reference^15^ describe in detail the data generation and quality control pipelines. Additional details on the recording sessions are provided in the Data Records section.

**Table 1:** List of experimental animals and their attributes.

Subject ID	Sex	Date of Birth (YYYYMMDD)	Imaged Cell Type	Imaging Plane
408021	M	20180623	L2/3 Pyr	somata
411400	F	20180711	L5 Pyr	distal apical dendrites
411424	F	20180711	L2/3 Pyr	somata
411771	M	20180713	L5 Pyr	somata
412933	M	20180718	L2/3 Pyr	distal apical dendrites
413663	M	20180721	L2/3 Pyr	distal apical dendrites
418779	F	20180820	L5 Pyr	somata
420011	F	20180826	L5 Pyr	distal apical dendrites
433414	F	20181106	L2/3 Pyr	distal apical dendrites
433448	M	20181106	L5 Pyr	distal apical dendrites
433451	M	20181106	L5 Pyr	distal apical dendrites
433458	M	20181106	L5 Pyr	somata
440889	F	20181212	L2/3 Pyr	somata

**Table 2:** List of imaging sessions and their attributes.

Subject ID	Session ID	Imaging Date	Depth (*μ*m)	# ROIs	# Tracked ROIs	QC	Stimulus Seed
408021	758519303	20180926	175	96	59	passed	30587
408021	759189643	20180927	175	74	59	passed	5730
408021	759660390	20181001	175	107	59	passed	36941
411400	759666166	20181001	20	942	0	failed	11883
411400	759872185	20181002	20	289	0	failed	8005
411400	760269100	20181003	20	524	0	failed	34380
411400	761730740	20181009	20	630	162	passed	44023
411400	762415169	20181011	20	637	162	passed	29259
411400	763646681	20181015	20	597	162	passed	1118
411424	761624763	20181009	175	87	55	passed	997
411424	761944562	20181010	175	90	55	passed	33856
411424	762250376	20181011	175	80	55	passed	23187
411771	760260459	20181003	375	90	47	passed	33767
411771	760659782	20181004	375	70	47	passed	32698
411771	761269197	20181008	375	79	47	passed	17904
412933	763949859	20181016	75	1041	0	failed	44721
412933	764897534	20181017	75	948	0	failed	32579
412933	765427689	20181018	75	836	0	failed	26850
412933	766755831	20181022	50	344	98	passed	39002
412933	767254594	20181023	50	168	98	passed	6698
412933	768807532	20181025	50	250	98	passed	8612
413663	764704289	20181017	50	628	136	passed	12470
413663	765193831	20181018	50	348	136	passed	7038
413663	766502238	20181022	50	512	136	passed	23433
418779	777496949	20181112	375	15	12	passed	32706
418779	778374308	20181113	375	26	12	passed	8114
418779	779152062	20181114	375	29	12	passed	11744
420011	777914830	20181113	20	205	51	passed	20846
420011	778864809	20181114	20	159	51	passed	35159
420011	779650018	20181115	20	182	51	passed	34931
433414	826187862	20190220	75	727	118	passed	303
433414	826773996	20190221	75	300	118	passed	13515
433414	827833392	20190222	75	333	118	passed	32899
433448	826338612	20190220	20	1636	112	passed	38171
433448	826819032	20190221	20	445	112	passed	38273
433448	828816509	20190225	20	496	112	passed	18246
433448	829283315	20190226	20	436	0	passed	17769
433451	823453391	20190215	20	966	353	passed	18665
433451	824434038	20190218	20	1029	353	passed	36
433451	825180479	20190219	20	986	353	passed	7754
433458	826659257	20190221	375	99	70	passed	35969
433458	827300090	20190222	375	87	70	passed	10378
433458	828475005	20190225	375	97	70	passed	10576
433458	829520904	20190227	375	88	0	passed	42270
440889	832883243	20190306	175	224	147	passed	27797
440889	833704570	20190307	175	224	147	passed	16745
440889	834403597	20190308	175	210	147	passed	10210
440889	836968429	20190314	175	205	0	passed	24253
440889	837360280	20190315	175	217	0	failed	19576
440889	838633305	20190318	175	227	0	failed	30582

Data were collected and processed using the Allen Brain Observatory data collection and processing pipelines^15^. Imaging was performed with Nikon A1R MP + two-photon microscopes equipped with 16X Nikon water dipping objectives (N16XLWD-PF). Laser excitation was provided at a wavelength of 910 nm by a Ti:Sapphire laser (Chameleon Vision-Coherent). Calcium fluorescence movies were recorded at 30 Hz with resonant scanners over a 400 *μ*m field of view with a resolution of 512 × 512 pixels (see Video 1, deposited on FigShare^16^). Temporal synchronization of calcium imaging, visual stimulation, running disc movement, and infrared pupil recordings was achieved by recording all experimental clocks on a single NI PCI-6612 digital IO board at 100 kHz. Neuronal recordings were motion corrected, and ROI masks of neuronal somata were segmented as described previously^15^.

For recordings in layer 1, ROI masks of neuronal dendrites were segmented using the robust estimation algorithm EXTRACT^17,18^ (https://github.com/schnitzer-lab/EXTRACT-public), which allows non-somatic shaped ROIs to be identified. The parameters used with EXTRACT are described next. First, the motion-corrected recordings were high-pass filtered spatially (spatial_highpass_cutoff = 10) and downsampled temporally to 15 Hz (downsample_time_by = 2). The algorithm was set to enable spatially discontinuous dendritic segments to be identified as part of single ROIs (dendrite_aware = True). Once putative ROIs had been identified, the following inclusion parameters were applied: (1) minimum peak spatial SNR of 2.5 (cellfind_min_snr = 2.5), (2) minimum temporal SNR of 5 (T_min_snr = 5), and (3) maximum spatial corruption index of 1.5 (spatial_corrupt_thresh = 1.5). Details of the parameter definitions can be found in the EXTRACT GitHub repository^18^. For all other EXTRACT parameters, the default settings were used.

Following segmentation, fluorescence traces for both somatic and dendritic ROIs were extracted, neuropil-subtracted, demixed, and converted to ΔF/F traces, as described previously^15,19^. Together, neuropil subtraction and the use of a 180-second (5401 sample) sliding window to calculate rolling baseline fluorescence levels (F) for the ΔF/F computation ensured that the ΔF/F traces obtained were robust to potential differences in background fluorescence between mice and imaging planes. Finally, any remaining ROIs identified as being duplicates or unions, overlapping the motion border or being too noisy (defined as having a mean ΔF/F below 0 or a median ΔF/F above the mid-range ΔF/F, i.e., the midpoint between the minimum and maximum) were rejected. In the somatic layers, 15–224 ROIs per mouse per session were identified and retained for analysis, compared to 159–1636 ROIs in the dendritic layers. Lastly, maximum-projection images were obtained for each recording, examples of which are shown in Figs. 1b, 2b. Briefly, the motion corrected recordings were downsampled to ~4 Hz by averaging together every 8 consecutive frames, following which the maximum value across downsampled frames was retained for each pixel. The resulting images were then rescaled to span the full 8-bit pixel value range (0–255).

### Visual stimulation

During each habituation and imaging session, mice viewed both a Gabor sequence stimulus and a visual flow stimulus. The stimuli were presented consecutively for an equal amount of time and in random order. They appeared on a grayscreen background and were projected at 60 Hz on a flat 24-inch monitor positioned 10 cm from the right eye. The monitor was rotated and tilted to appear perpendicular to the optic axis of the eye, and the stimuli were warped spatially to mimic a spherical projection screen. Whereas habituation sessions increased in duration over days from 10 to 60 minutes, optical imaging sessions always lasted 70 minutes, comprising 34 minutes of Gabor sequence stimulus and 17 minutes of visual flow stimulus in each direction. Each stimulus period was flanked by 1 or 30 seconds of grayscreen for the habituation and optical imaging sessions, respectively.

**Fig. 3:**
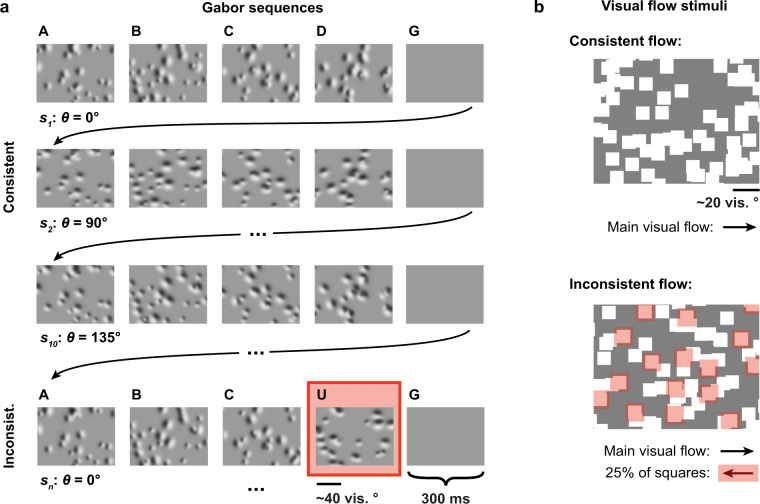
(**a**) Illustration of the Gabor stimuli presented to the mice. The red box marks the inconsistent occurrence of a *U* image. (**b**) Illustration of the visual flow stimuli presented to the mice. The red boxes mark squares moving in the opposite direction to the main flow.

The Gabor sequence stimulus was adapted from a previously published study^20^. Specifically, it consisted of repeating 1.5-second sequences, each comprising five consecutive images (*A-B-C-D-G*) presented for 300 ms each. Whereas *G* images were uniformly gray, images *A*, *B*, *C*, and *D* were defined by the locations and sizes of the 30 Gabor patches they each comprised. In other words, throughout a session, the locations and sizes of the Gabor patches were the same for all *A* images, but differed between *A* and *B* images, etc. Furthermore, these locations and sizes were always resampled between mice, as well as between days, such that no two sessions comprised the same Gabor sequences, even for the same mouse. The location of each Gabor patch was sampled uniformly over the visual field, while its size was sampled uniformly from 10 to 20 visual degrees. Within each repeat of the sequence (*A-B-C-D-G*), the orientations of each of the Gabor patches were sampled randomly from a von Mises distribution with a shared mean and a *κ* (dispersion parameter) of 16. The shared mean orientation was randomly selected for each sequence and counterbalanced for all four orientations {0°, 45°, 90°, 135°}. As such, although a large range of Gabor patch orientations were viewed during a session, orientations were very similar within a single sequence. “Inconsistent” sequences were created by replacing *D* images with *U* images in the sequence (*A-B-C-U-G*). *U* images differed from *D* images not only because they were defined by a distinct set of Gabor patch sizes and locations, but also because the orientations of their Gabor patches were sampled from a von Mises distribution with a mean shifted by 90° with respect to the preceding regular images (*A-B-C*), namely from {90°, 135°, 180°, 225°} (Fig. 3a, and Video 2 on FigShare^16^).

The visual flow stimulus consisted of 105 white squares moving uniformly across the screen at a velocity of 50 visual degrees per second, with each square being 8 by 8 visual degrees in size. The stimulus was split into two consecutive periods ordered randomly, and each defined by the main direction in which the squares were moving (rightward or leftward, i.e., in the nasal-to-temporal direction or vice versa, respectively). Inconsistent sequences, or flow violations, were created by reversing the direction of flow of a randomly selected 25% of the squares for 2–4 seconds at a time, following which they resumed their motion in the main direction of flow (Fig. 3b, and Video 3 on FigShare^16^).

Inconsistent sequences, accounting for approximately 7% of the Gabor sequences and 5% of visual flow stimulus time, *only* occurred on optical imaging days, and not on habituation days. In particular, each 70-minute imaging session was broken up into approximately 30 blocks, each comprising 30–90 seconds of consistent sequences followed by several seconds of inconsistent sequences (3–6 seconds for Gabor sequence stimulus and 2–4 seconds for the visual flow stimulus). All durations were sampled randomly and uniformly for each block, across multiples of 1.5 seconds for the Gabor sequence stimulus and of 1 second for the visual flow stimulus. See the Code Availability section for details on where to find the code to reproduce these stimuli.

### Running and pupil tracking

**Fig. 4:**
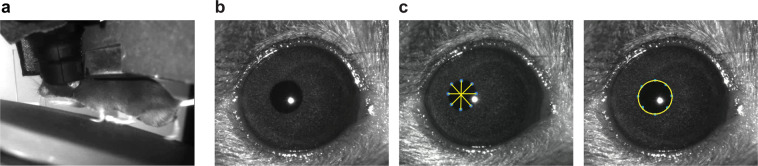
(**a**) Example image of a mouse on a running disc, under the two-photon microscope objective. (**b**) Example image of a mouse pupil. (**c**) (*left*) Example of a pupil in which 8 poles have been labelled using DeepLabCut to allow pupil position (average position of the 8 poles) and diameter (average length of the 4 diameters formed by the poles) to be estimated. (*right*) Inferred pupil ellipse.

Mice were allowed to run freely on a disc while head-fixed during habituation and optical imaging sessions (Fig. 4a, and Video 4 on FigShare^16^). Running information was collected at 60 Hz and converted from disc rotations per running frame to cm/s. The resulting velocities were median-filtered with a five-frame kernel size, and any remaining outliers, defined as resulting from a single frame velocity change of at least ±50 cm/s, were omitted from analyses.

To track pupil position and diameter during imaging sessions, an infrared LED illuminated the eye ipsilateral to the monitor (right eye), allowing infrared videos to be recorded (Fig. 4b, and Video 5 on FigShare^16,21^). We trained a DeepLabCut model from ~200 manually labelled examples to automatically label points around the eye, from which we estimated the pupil diameter and centroid position (~0.01 mm per pixel conversion)^22^ (Fig. 4c,d). For the pupil centroid position, data for each label is stored as pupil_position_x, pupil_position_y, which indicate the horizontal and vertical distances, respectively, in mm from the top-left corner of the pupil recording videos. When analysing pupil diameter traces, we omitted outlier frames, defined as resulting from a single-frame diameter change of at least 0.05 mm, which usually reflected blinking.

### ROI tracking across sessions

To track ROIs across days, we employed a custom-modified version of the ROI-matching package developed to track cell bodies across multiple recording days by the Allen Institute for Brain Science^15^. This pipeline implements the enhanced correlation coefficient image registration algorithm to align ROI masks, and the graph-theoretic blossom algorithm to optimize the separation and degree of overlap between pairwise matches, as well as the number of matches across all provided sessions^23^. This process produced highly plausible matches for the somatic ROIs. However, it provided some implausible matches for the smaller and more irregularly shaped dendritic ROIs. For the dendritic ROIs, we therefore further constrained the putative matches to those that overlapped by at least 10–20%. Finally, we merged results across all session orderings (e.g., 1-2-3, 1-3-2, 3-1-2), eliminating any conflicting matches, i.e., non-identical matchings that shared ROIs. In total, the modified matching algorithm produced ~100–500 highly plausible matched ROIs per plane, i.e., ~32–75% of the theoretical maximum number of trackable ROIs (L2/3-D: *n* = 254, L2/3-S: *n* = 261, L5-D: *n* = 516, L5-S: *n* = 129) (Fig. 2b,c).

## Data Records

The full dataset is publicly available in the Neurodata Without Borders (NWB) format^24^ on the DANDI Archive (https://dandiarchive.org/dandiset/000037)^13^. In addition, illustrative videos with example calcium imaging, stimulus, and behavioural recordings are available on FigShare^16^.

### Data organization

The dataset is organized as follows on the DANDI Archive. The files for the 50 total sessions recorded are organized by subject into folders. For example, files for sessions recorded in subject 408021 are stored in folder sub-408021. Within the folders, each file contains data for a single recording session. Notably, however, we created three versions of each session file, each with increasingly more data included. The versions are the basic version [B], the version with the stimulus frame images [I], and the version with the motion corrected two-photon calcium imaging stack [S]. The contents of the files are as follows:

1. ROI ΔF/F traces [B, I, S]
2. ROI masks [B, I, S]
3. ROI tracking indices, for tracked sessions [B, I, S]
4. Recording plane image [B, I, S]
5. Running velocity traces [B, I, S]
6. Pupil diameter traces [B, I, S]
7. Pupil centroid position traces [B, I, S]
8. Detailed stimulus parameter table [B, I, S]
9. Stimulus frame images [I, S]
10. Motion corrected two-photon calcium imaging stack [S]

The multiple versions were created under the expectation that most users will only need the data contained in the basic version [B], amounting to about 130 MB to 1.7 GB per file. Adding the stimulus frame images increased the file sizes by about 1.5 GB each [I]. Further adding the motion corrected imaging stack increased the file sizes much more, by about 45 GB per file [S]. Although NWB files on the DANDI Archive can be accessed remotely and streamed, we anticipated that the added data could create a substantial burden in terms of both bandwidth and storage for users wishing to download the dataset and use it locally.

The naming convention for the three versions on DANDI is as follows: sub-{unique subject ID}_ses-{unique session ID}_{content}.nwb, where:

1. B (basic): content = behavior + ophys, e.g., sub-408021_ses-758519303_behavior + ophys.nwb
2. I (with stimulus images): content = behavior + image + ophys, e.g., sub-408021_ses-758519303_behavior + image + ophys.nwb
3. S (with motion corrected imaging stack): content = obj-raw_behavior + image + ophys, e.g., sub-408021_ses-758519303_obj-raw_behavior + image + ophys.nwb

### Animal and recording session attributes

As noted above, data for 50 recording sessions total were gathered from 13 animals. Of these, two animals had at least one session that did not meet the Allen Institute’s previously-described^15^ quality control thresholds, and could therefore be considered for exclusion from analysis. In addition, for some animals, more than three imaging sessions were collected, for example if an early session had not passed quality control thresholds. We note that, due to including recordings from 4 distinct imaging planes, there may be an insufficient number of animals to perform robust splits of some cohorts. For example, while the dataset is well-split between male (7) and female (6) subjects, splitting the data further by sex may result in some groups with *N* = 1 (e.g., there is only 1 female L2/3-D mouse). Table 1 summarizes all of the experimental subjects. For each animal, the following information is provided: **(1) Subject ID:** unique ID assigned to the animal (6 digits), **(2) Sex:** subject’s sex, **(3) Date of Birth:** subject’s date of birth in the YYYYMMDD format, **(4) Imaged Cell Type:** the type of cell in which imaging was performed, i.e., either layer 2/3 pyramidal neurons (L2/3 Pyr) or layer 5 pyramidal neurons (L5 Pyr), and **(5) Imaging Plane:** the cortical plane in which two-photon calcium imaging was performed, i.e., either the plane in which the cell bodies are located (somata) or the plane in which the distal apical dendrites are located.

Table 2 summarizes all of the imaging sessions, with the following information provided: **(1) Subject ID:** unique ID assigned to the animal (6 digits), **(2) Session ID:** unique ID assigned to the recording session (9 digits), **(3) Imaging Date:** date on which imaging was performed in the YYYYMMDD format, **(4) Depth (*μ*m):** cortical depth to which the imaging was targeted, in *μ*m, **(5) # ROIs:** total number of ROIs segmented for the session, **(6) # Tracked ROIs:** number of ROIs tracked across sessions for the subject (0 for sessions that were not included in the tracking), **(7) QC:** whether the session passed the Allen Institute’s quality control thresholds, and **(8) Stimulus Seed:** the random number generator seed used to generate the stimuli for the session.

Additional notes on the imaging sessions are included in the full metadata table (Supplementary Table 1, also available on the GitHub repository, https://github.com/jeromelecoq/allen_openscope_metadata/blob/master/projects/credit_assignement/metadata.csv. The table comprises the same columns as Tables 1, 2, with a few additional ones: **(1) Dandiset:** the DANDI dataset number (000037), **(2) Local Subject #:** the subject number within the dataset (1–13), **(3) Local Session #:** the session number for the subject (1–6), **(4) Imaging Date and Time (UTC):** the imaging start date and time in the UTC time zone, in the YYYYMMDDTHHMMSS format, **(5) Imaging Age (Weeks)**: age of the subject in weeks at the time of imaging, and **(6) Experimental Notes:** Any experimental notes recorded for the session.

### Overview of data

**Fig. 5:**
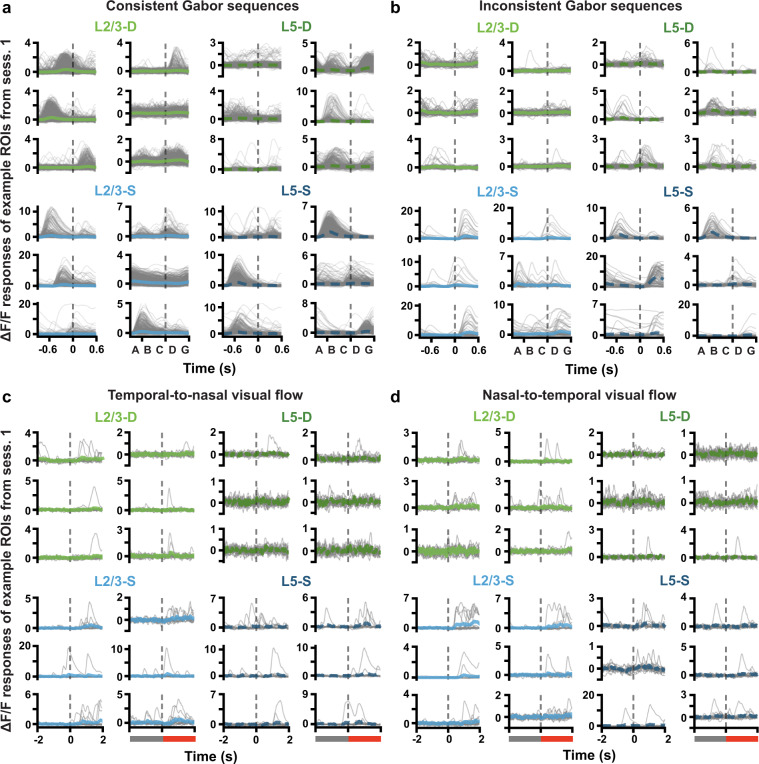
(**a**) ΔF/F response traces to each consistent Gabor sequence (gray) for example ROIs. Mean (±SEM) ΔF/F responses across sequences are plotted in blue or green. Dashed vertical lines mark onset of *D* images. Plotted ROIs were randomly selected from session 1 ROIs deemed consistently responsive to Gabor sequences, based on the following criteria: (1) their SNR was above the median for the session, (2) the median pairwise correlation between their individual sequence responses, as well as the standard deviation and skew of their mean response, were each above the 75^*th*^ percentile for the session. Responses to individual sequences were smoothed using a four-point moving average, for correlation calculation and plotting, only. (**b**) Same as a., but for inconsistent sequences. (**c**) ΔF/F response traces to the onset of inconsistent flow, during temporal-to-nasal visual flow. Dashed vertical lines mark onset of inconsistent flow at time 0. Plotted ROIs were randomly selected from session 1 ROIs deemed responsive to the onset of inconsistent visual flow, based on the following criteria: (1) their SNR was above the median for the session, (2) the median pairwise correlation between their individual sequence responses to inconsistent flow, as well as the difference in mean response to inconsistent vs consistent flow, were each above the 75^*th*^ percentile for the session. (**d**) Same as c., but for nasal-to-temporal visual flow.

To provide some intuition for the nature of the data, we present here population-wide responses to the stimuli over days, and a brief example of the behavioural data. As this is a data descriptor paper, we leave aside any statistical analyses and interpretations, and only present an overview of the fluorescence signals observed, using some randomly selected examples. Both the somatic and dendritic ROIs showed clear responses to both the Gabor and visual flow stimuli, with many showing increased fluorescence responses to the onset of the stimuli (Fig. 5). There were also clear differences in the responses to the consistent versus inconsistent stimuli as well (Fig. 5a versus b, and c & d).

**Fig. 6:**
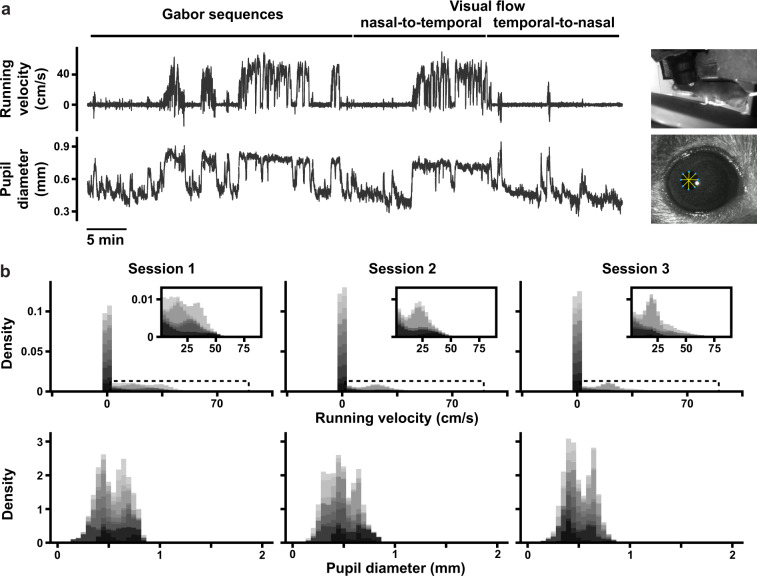
(**a**) (*Top*) Example running velocity trace (in cm/s) and (*bottom*) pupil diameter trace (in mm) for an example optical physiology session, lasting 70 min. Stimulus blocks are shown at the top, and the scale bar shows 5 min. (**b**) Histograms of aggregated running velocities (*top*) and pupil diameters (*bottom*), for each session. Data is stacked across all mice (11), with each mouse represented by a different shade of gray. Insets for the running velocity histograms (*top*) show close-ups of the areas marked by the dotted rectangles.

With respect to the behavioural data, we provide plots showing the raw behavioural signal in an example mouse (Fig. 6a) and distributions of the signals across recording sessions, aggregated across mice (Fig. 6b). These records can enable analyses of the behavioural changes (if any) induced by the different stimuli.

## Technical Validation

In the dataset, we provide the pre-processed fluorescence responses of the spatial ROIs (cell bodies or distal apical dendrite segments, depending on the imaging plane) segmented from our microscopy recordings. These data were included in addition to the raw calcium imaging files, because most analyses of two-photon calcium imaging data focus on extracted ROI activity traces, and they are much more compact than the raw imaging data. As described above, raw fluorescence traces are extracted for each ROI, and then baselined using a sliding window to obtain a measure of change in fluorescence relative to baseline, i.e., a ΔF/F. There are several steps to the pre-processing that we validate here, including the stability and quality of the microscopy, the quality of the segmentation, and the ability to match the ROIs across days.

**Fig. 7:**
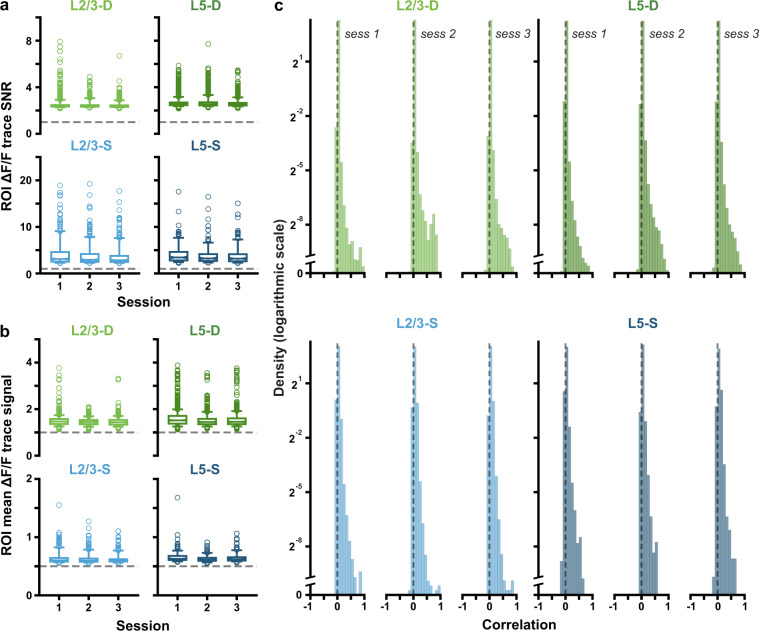
(**a**) ΔF/F trace SNRs for each ROI. For each session and plane, boxplots show the medians of the ROI SNR distributions, as well as the 25^*th*^ to 75^*th*^ percentiles, with the whiskers extending from the 5^*th*^ to 95^*th*^ percentiles. Dashed horizontal lines mark 1, i.e., noise level. (**b**) Mean ΔF/F trace signal, where each datapoint corresponds to an ROI. Boxplots drawn as in a., and signal is the mean activity above the noise threshold used to calculate SNR. (**c**) Distributions of pairwise ROI correlations, plotted on a log scale. The log scale is linearized near 0, as signalled by the axis break, overemphasizing the lower tail for visibility. Pairwise correlations were computed over full session fluorescence traces, which were smoothed using a four-point moving average.

To validate the quality and stability of our optical imaging data, we computed the SNR of each ROI in each recording session. SNR was computed as follows. First, the parameters (mean and standard deviation) of a normal distribution over noisy activity were estimated based on the lower half of each ROI’s full activity distribution. The 95^*th*^ percentile of the parameterized noise distribution was then defined as that ROI’s noise threshold. ROI SNRs were then calculated as the ratio between their mean activity above the noise threshold (signal), and the standard deviation of their parameterized noise distribution. These are shown in Fig. 7A, and demonstrate that our recordings have relatively high SNR (>1) and that this is quite stable over days. Similarly, the mean ΔF/F signal was stable over days (Fig. 7b).

In assessing the reliability of the ROI segmentation, we were mostly concerned that the algorithm identifying the ROIs could over-segment the apical dendrites, yielding multiple ROIs that are, in fact, part of the same dendritic process. Segmenting the somata is much more straightforward because the somata are roughly circular in our imaging data and tend not to overlap (see, e.g., Fig. 2d). In contrast, the apical dendrite segments are elongated and often intersect with one another. If our algorithm were over-segmenting the branching apical dendrite structure, we would expect to see many pairs of highly-correlated dendrite ROIs (i.e., pairs of ROIs that are actually part of the same dendritic process). Thus, to validate the segmentation we computed the correlation of the ΔF/F traces for each pair of ROIs in each recording. The distributions of correlation coefficients were very similar for the apical dendrite ROIs and for the somatic ROIs (Fig. 7c), suggesting that we were unlikely to be heavily over-segmenting the dendritic data. Instead, the high number of dendritic segments identified in many planes likely include many independently active segments of the same neurons and dendrites vertically traversing the imaging planes. To be more conservative, ROIs with correlations above 0.8 (e.g., approximately 0.01% of possible pairs of L2/3 dendrites) or those with similar trial-averaged visual stimulus-triggered responses could be merged. The raw data is available for independent segmentation and analysis.

**Fig. 8:**
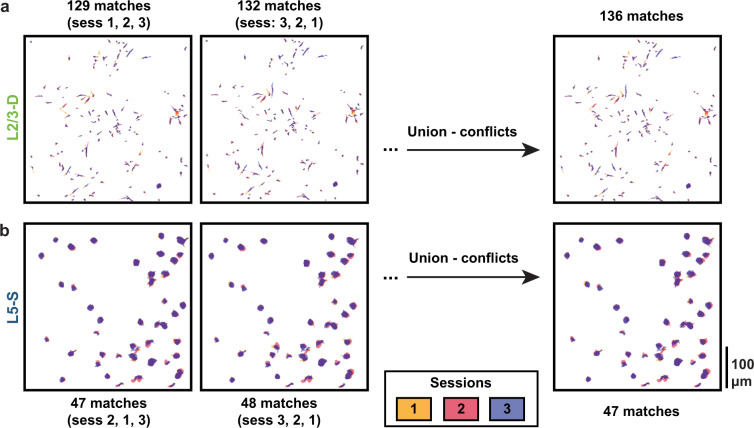
(**a**) Example L2/3-D mouse with ROIs matched across sessions. The order in which the session images are aligned slightly affects which ROIs are matched. (*Left*) Permutation with the smallest number of matched ROIs. (*Middle*) Permutation with the largest number of matched ROIs. (*Right*) Taking the union of matches across all session permutations while removing conflicting matches (matches comprising at least one ROI that also appears in a different match) enables the quantity and quality of matched ROIs to be optimized. In this example, four pairwise matches were identified as conflicts and removed, yielding 136 final matches. (**b**) Same as a., but for a L5-S mouse. The variation in number of matched ROIs across session orderings for somata was generally far less than that for dendrites due to their larger sizes and more regular shapes. Combining matched ROIs across all permutations did nonetheless, in this example mouse, enable two of the pairwise matches to be identified as conflicts and removed, yielding 47 final matches.

One valuable aspect of our dataset is that we image the same fields of view over multiple days, enabling us to track how individual ROIs change their responses over days. This requires that ROIs be matched across days, in order to identify which ROI ID in one day’s recording matches a given ROI ID in another day’s recording. This can be very challenging, as it requires being able to find the exact same plane, in all 3 dimensions, at each recording session. Even if this is done successfully, the segmentation routine is not guaranteed to identify the same ROIs (or even the same number of ROIs) in each recording session. Lastly, the outcome of the ROI matching routine depends to some degree on the order in which it receives the different sessions’ ROI masks. For this reason, we repeated the ROI matching using all possible permutations of session ordering, and then used the union of the set of matches (over permutations) minus the conflicts (matches comprising at least one ROI that also appears in a different match within another permutation) as our putative ROI matches. Figure 8 shows the ROI matches from an example set of apical dendrite recordings (3 sessions), and from an example set of somatic recordings (3 sessions). The ROI masks from each session overlap substantially in the merged image, reflecting the consistency of our imaging planes and reliability of our ROI matching procedure.

**Fig. 9:**
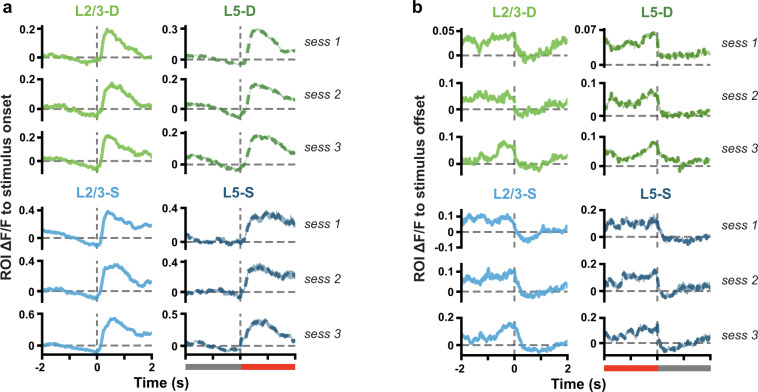
(**a**) Mean (±SEM) ΔF/F response traces across ROI mean responses to stimulus onset (Gabor sequence) from grayscreen. Dashed vertical line at time 0 marks stimulus onset, also signalled by the gray bar becoming red (bottom of right column). (**b**) Same as a., but for stimulus offset. Dashed vertical line at time 0 marks stimulus offset, as signalled by the red bar becoming gray (bottom of right column).

Finally, to validate that our stimuli are temporally well-aligned with our neural recordings and that the calcium signal is tracking visually evoked responses, we computed the mean ΔF/F in the time windows surrounding the stimulus onsets (transition from gray screen to Gabor sequences or visual flow) and offsets (transition from Gabor sequences or visual flow to gray screen). These ΔF/F traces show distinct transients that align with the stimulus onsets and offsets (Fig. 9), validating our temporal alignment, and demonstrating clear stimulus responses in the identified ROIs.

## Usage Notes

For users with experience using the NWB data format who are interested in running their own analyses from scratch, the dataset can be downloaded directly from the DANDI Archive and inspected using tools like PyNWB if using Python, and MatNMB, if using MATLAB^24^. As described above, 50 sessions were recorded across the mice, and for each session, three files are available for download. The file versions with only the basic data range in size from 130 MB to 1.7 GB. If only the basic data files for sessions 1 to 3 that passed quality control are needed, the total download size is approximately 15 GB for 33 files in total. For users wishing to work with the stimulus images as well, the file versions that also include the stimulus frame images range in size from 1.5 to 3.1 GB each. Lastly, the file versions that also include the full motion corrected two-photon calcium imaging stack are approximately 45 GB each. These may be useful, for example, for users wishing to deploy their own segmentation and ΔF/F conversion pipelines on our data. They can also be used to compute statistics for converting raw fluorescence to photons, if desired^25^. The following notebook on GitHub provides example code for computing photon gain and offset directly from raw imaging stacks: https://github.com/jeromelecoq/QC_2P/blob/master/Example%20use%20of%20QC_2P.ipynb. Lastly, although running velocity, pupil diameter and pupil centroid position are provided in the data files, other behavioural metrics like direction of gaze were not computed for this dataset. For users wishing to work with this type of data, behaviour and pupil recording videos (see Fig. 4) are available upon request to the corresponding author.

For users wishing to work with existing code, detailed resources for analysing and exploring this specific dataset in Python are provided in a GitHub repository (https://github.com/colleenjg/OpenScope_CA_Analysis). Users can install the conda environment provided, following the instructions in the README, and download specific sessions of interest. A few jupyter notebooks are provided for users to become familiar with the dataset. First, under examples, the session_demonstration_script.ipynb notebook provides users with step-by-step examples of how to load a file into a custom Python object, i.e. the Session object, and to plot average stimulus responses for individual ROIs, retrieve ROI tracking information, and display ROI masks. Second, a jupyter notebook is provided under minihack called mini_hackathon.ipynb which provides examples of various analyses users could be interested in running on the data. Lastly, in the main directory, the run_paper_figures.ipynb notebook shows how the codebase can be used to reproduce the figures presented here directly on the dataset.

## Supplementary information

Supplementary Table 1

## Data Availability

Data pre-processing was performed in Python 3.6^26^ with custom scripts that are freely available on GitHub (https://github.com/colleenjg/OpenScope_CA_Analysis) and were developed using the following packages: NumPy^27^, SciPy^28^, Pandas^29^, Matplotlib^30^, Scikit-learn 0.21.1^31^, and the AllenSDK 1.6.0. (https://github.com/AllenInstitute/AllenSDK). Stimuli were generated by Python 2.7^32^ custom scripts based on PsychoPy 1.82.01^33^ and CamStim 0.2.4. The code is freely available (along with instructions to reproduce the stimuli, and example videos) on GitHub (https://github.com/colleenjg/cred_assign_stimuli). Dendritic segmentation was run in Matlab 2019a using a robust estimation algorithm^17,18^ (https://github.com/schnitzer-lab/EXTRACT-public). Pupil tracking was performed using DeepLabCut 2.0.5^22^ (http://www.mackenziemathislab.org/deeplabcut). ROIs were matched across sessions using a custom-modified version of the n-way cell matching package developed by the Allen Institute (https://github.com/AllenInstitute/ophys_nway_matching). Code for estimating photon conversion statistics on the raw imaging stacks is available on GitHub^25^ (https://github.com/jeromelecoq/QC_2P/blob/master/Example%20use%20of%20QC_2P.ipynb).
